# Eastern Equine Encephalitis Virus: The Importance of Metabolism and Aging

**DOI:** 10.3390/ijms252413318

**Published:** 2024-12-12

**Authors:** Pawel Kordowitzki

**Affiliations:** 1 Department of Preclinical and Basic Sciences, Faculty of Biological and Veterinary Sciences, Nicolaus Copernicus University, 87-100 Torun, Poland; p.kordowitzki@umk.pl; 2 Department of Cell Biology, Harvard Medical School, Boston, MA 02115, USA

**Keywords:** eastern equine encephalitis virus, very low-density lipoprotein receptor, NLRP3 inflammasome

## Abstract

Eastern equine encephalitis virus (EEEV) is a mosquito-transmitted alphavirus that, among humans, can cause a severe and often fatal illness. The zoonotic EEEV enzootic cycle involves a cycle of transmission between *Culiseta melanura* and avian hosts, frequently resulting in spillover to dead-end vertebrate hosts such as humans and horses. Interestingly, it has been described that the W132G mutation of the very low-density lipoprotein receptor (VLDLR), the receptor of EEEV, significantly enhanced the VLDLR-mediated cell attachment of EEEV. The patient’s metabolism plays a pivotal role in shaping the complex landscape of viral zoonosis. EEEV represents a significant public health concern due to its severe clinical outcomes, challenging epidemiological characteristics, and certain risk factors that heighten susceptibility among specific populations or age groups. Age is one of several predictors that can impact the outcome of EEEV infection; juvenile animals appear to be particularly vulnerable to severe disease. This has also been observed in natural infections, as children are often the most severely impacted humans. The aim of this piece is to shed light on the intricate relationship between human metabolism and the Eastern equine encephalitis virus.

## 1. Introduction

**Figure 1:**
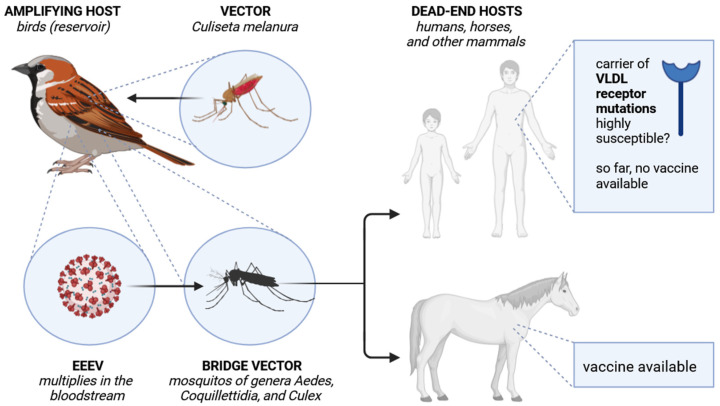
The scheme shows the Eastern equine encephalitis virus (EEEV) transmission.

Eastern equine encephalitis is considered a rare but severe viral disease caused by the Eastern equine encephalitis virus (EEEV) [1]. EEEV presents a notable epidemiological profile characterized by its transmission through mosquito vectors and certain risk factors that heighten susceptibility among specific populations. The EEEV is a member of the family *Togaviridae*, genus *Alphavirus*, which is transmitted via the bite of an infected mosquito and essentially causes disease in mammals, especially horses and humans (Figure 1) [1]. The EEEV genome is about 11 kb in size, containing four nonstructural proteins, namely the nsP1, nsP2, nsP3, and the nsP4, and five structural proteins such as the Capsid, E3, E2, 6K, and the TF protein [2]. The major vectors for the excursive strain are *Culiseta melanura*, together with *Ornithophagus kalmia* and *Coquillettidia perturbans*, which participate in the transmission of the epizootic strain. There are two EEEV strains: the epizootic/epidemic strain, which cycles between vectors and bridging vector-competent birds and can infect mammalian hosts, and the enzootic strain, which cycles between *Culiseta melanura* and *Haemagogus youngi*. The latter cross-infects birds in the presence of *Culiseta melanura* but results in low titer viremia and does not reach infective viremia proportions [1,2,3]. EEEV virus represents a significant public health concern due to its severe clinical outcomes, such as severe neurological symptoms, and challenging epidemiological characteristics as recent cases in the United States of America have increased [2]. In addition, early symptoms like fever and headache are non-specific; therefore, the diagnosis is challenging. Critical for the pathogenesis of the disease is the binding of EEEV to the very low-density lipoprotein receptor (VLDLR), which will be described in detail in Section 2. The EEE virus presents unique challenges in disease transmission control and risk assessment [3]. Geographically, the virus is so far most prevalent in the United States, in the Atlantic and Gulf Coast states, and in parts of the Great Lakes region [4]. Climate and environmental factors, such as changes in precipitation, temperature, and humidity, can influence mosquito population dynamics, breeding patterns, and virus transmission rates. Variations of the aforementioned climatic conditions significantly influence the abundance and distribution of mosquito vectors, consequently affecting the prevalence and spread of Eastern equine encephalitis virus in endemic regions [4,5]. Its transmission peaks during late summer and early fall, aligning with mosquito breeding patterns, underscoring the critical need for effective vector control strategies during these times [1,3]. 

The patient’s metabolism, meaning the complex network of biochemical interactions that sustain life, plays a critical role in shaping the complex landscape of viral zoonosis [6]. The dynamic and finely-tuned connection between metabolic programs and the specialized cellular functions they support has emerged as a crucial factor in understanding the immune system’s response to viral infections. Based on contemporary research, there are different ways in which viruses can manipulate the host cell’s metabolism with the aim of creating an environment favorable to their proliferation and survival [6]. In this regard, numerous viruses have evolved pathways to take over the host’s metabolic pathways, diverting the flow of energy and resources towards the synthesis of viral genetic material and proteins. It is worth mentioning that due to the aforementioned strategy, necessary building blocks for viral replication are provided, and moreover, the host’s defense mechanisms are suppressed, enabling the virus to evade immune surveillance and establish a successful infection. Interestingly, mitochondria, also known as the so-called powerhouses of the cell [7], have arisen as a key player in the critical cross-talk between metabolism and viral infections [8]. 

Mitochondria are not only central to energy production but also contribute to the activation and regulation of immune signaling pathways. Notably, viruses have been shown to interact with the host cell mitochondria, leading to a disruption of their structure and function, which, in consequence, can have far-reaching outcomes on the host’s immune response [9]. Due to the disruption of mitochondrial dynamics, in other words, creating imbalances between mitochondrial fission and fusion, viruses are able to promote their infection and survival [10]. This mitochondrial dysfunction can lead to the accumulation of damaged mitochondria, triggering apoptosis and compromising the host’s ability to react with an effective immune response. Furthermore, upon the suppression of mitophagy, the imbalance in mitochondrial dynamics is exacerbated, leading to the creation of an intracellular environment that is favorable for viral proliferation [11]. The management of EEEV infection is characterized by a low number of available therapeutic strategies, which are mainly based on supportive care, necessitating robust public health strategies focused on prevention. These factors highlight the importance of mosquito control and public education in preventing EEEV. The aim of this piece is to shed light on the intricate relationship between human metabolism and the Eastern equine encephalitis virus to encourage further research on human vaccines, which is an ultimate need.

## 2. Eastern Equine Encephalitis Virus and the Very Low-Density Lipoprotein Receptor

As a pathogen primarily transmitted by mosquito vectors (Figure 1), EEEV presents unique challenges in disease transmission control and risk assessment. The epidemiological landscape of EEEV is shaped by vectors and specific risk factors, including enhanced susceptibility in immunocompromised populations, especially in organ transplant recipients [12]. Interestingly, in the recent publication by Cao and coworkers [13], it has been described that the W132G mutation of the very low-density lipoprotein receptor (VLDLR), the receptor of EEEV [14], significantly enhanced the VLDLR-mediated cell attachment of EEEV, especially in the strains EEEV PE6 and PE6-K206E VLPs. The mutation W132G has been detected in the human genome, but the impact on the patient’s susceptibility remains elusive [13]. In consequence, it might be helpful to screen for the mentioned mutation in human patients to elucidate if carriers of this VLDL receptor variant are more susceptible to an EEEV infection. Other low-density lipoprotein receptors, including the LDLRAD3 and the apolipoprotein E receptor 2 (ApoER2), have been described as entry receptors for different alphaviruses in general. Noteworthy, ApoER2 was also identified as a receptor of EEEV. The EEEV invades host cells upon receptor-mediated endocytosis, during which the spikes of the E1-E2 heterodimers are responsible for the binding to the receptor [13]. Moreover, the limitation of relying on symptomatology to diagnose an EEEV infection necessitates the incorporation of advanced serological and molecular diagnostic methods, such as the detection of EEEV RNA and IgM antibodies. 

This being said, the detection of viral RNA in the absence of IgM antibodies is recommended as it indicates a recent EEEV infection. Moreover, it is worth it for transplant recipients to screen for VLDL receptor mutations [15]. However, so far, no human vaccine has been made available. Adequate protection from UV viremia was recently described in cynomolgus macaques [16]. In the equine species, the recommended and very protective vaccine against EEEV is an inactivated, concentrated, adjuvanted, tissue culture origin equine encephalomyelitis virus made with strain TC-83. The application of VLDLR mutation screening and mosquito control measures still remain fundamental cornerstones in high-risk areas [1,14]. 

In general, the role of the very low-density lipoprotein receptor in viral infection has been a topic of increasing interest in recent years. The VLDLR has been implicated in the pathogenesis of various viral diseases, including HIV infection [17,18]. One of the key mechanisms by which the VLDLR may contribute to viral infection is through its involvement in the innate immune response [19]. Viruses often employ strategies to evade or suppress the host’s innate immune defenses, and the VLDLR may play a role in this process. Innate immune cells, such as dendritic cells, express pattern recognition receptors that can recognize viral components, triggering antiviral responses [20]. Interestingly, recent studies provided evidence that the VLDLR can modulate the activity of the aforementioned pattern recognition receptors, potentially impacting the innate immune response to viral infection [20,21]. For example, the VLDLR has been found to interact with toll-like receptors, which are critical not only for the detection of viral nucleic acids but also for the induction of antiviral signaling pathways [20,22]. Furthermore, the VLDLR has been implicated in the regulation of inflammasome activation, which is an important mechanism for the production of proinflammatory cytokines in response to viral infection. This will be discussed in a more detailed fashion in another section below. 

## 3. Epidemiology and Clinical Manifestations of Eastern Equine Encephalitis Virus in Humans

The geographic distribution of the EEE virus is largely constrained to the United States, particularly in Atlantic and Gulf Coast states, as well as in some areas of the Great Lakes region [23]. Most human cases are from Florida, Georgia, Massachusetts, Louisiana, New York, North Carolina, and South Carolina [2,4,23]. It is further noted that several mosquito and bird species are known to have significant roles in the EEEV transmission cycle, especially those involved in the vector–bridge–vector cycle, and these must be considered. The incubation period of EEE clinical cases after exposure to EEEV is approximately 4–10 days [2]. Elevation of antibody titer occurs during the second week after exposure to the virus. Individuals infected with EEEV without showing explicit clinical symptoms usually become viremic. Increased titer at the initial stage after the onset of the illness suggests that the immune response prior to exposure to EEEV may be helpful in maintaining viral replication at higher levels [1,2,3].

The seasonal occurrence of EEE virus transmission typically peaks during the late summer and early fall, correlating with the breeding patterns of mosquito vectors. The role of mosquito vectors as primary transmitters of the EEE virus emphasizes the critical need for vector control measures, particularly during peak transmission seasons [24]. Populations at increased risk of EEE virus infection include individuals with compromised immune systems, who are especially vulnerable to donor-derived infections due to their suppressed immune responses [25]. This fact underscores the imperative need for stringent screening of organ donors to mitigate the risk of EEEV transmission through transplantation, necessitating vigilant monitoring and diagnostic evaluation in post-transplant care of respective patients. 

Diagnosing EEEV infection is specifically challenging since the disease’s clinical symptoms overlap with other forms of viral encephalitis. Therefore, the diagnostic procedure is particularly complex, given the subtlety of early symptoms. Advanced serological and molecular diagnostic tools aid in the identification of EEEV, especially through the detection of EEEV RNA, which is often more reliable than the presence of IgM antibodies in certain cases [26]. This emphasizes the importance of utilizing comprehensive diagnostic methods to accurately detect the virus. The clinical manifestations of Eastern equine encephalitis virus infection present a formidable challenge to healthcare providers, as these symptoms often mimic those of other viral encephalitides [27,28,29,30]. Patients typically present with a sudden onset of systemic symptoms such as fever and headache, which can rapidly progress to severe neuroinvasive disease, including encephalitis, characterized by confusion, seizures, and coma. The neurotropism of the EEE virus leads to high morbidity and mortality rates, emphasizing the clinical and public health importance of accurate and timely diagnosis [1,2]. This task is complicated by the symptom overlap with other viral infections, potentially delaying targeted therapeutic interventions and influencing patient outcomes negatively. Diagnostic challenges in EEE virus infection are further compounded by the difficulties of differentiating it from other causes of viral encephalitis using clinical criteria alone [31]. 

The limitation of relying on symptomatology necessitates the incorporation of advanced serological and molecular diagnostic methods to accurately identify EEE virus presence. These tests include the detection of EEEV RNA and IgM antibodies [15,25]. Notably, the detection of viral RNA in the absence of IgM antibodies is crucial, as it indicates a recent infection. Such insights highlight the critical role of molecular diagnostics in tracing the timeline of infection, which is essential for preventing virus transmission, especially in high-risk settings. The pursuit of effective diagnostic strategies is intertwined with the broader effort to manage and control EEE virus outbreaks. While serological and molecular tools are pivotal in individual diagnosis, their successful implementation can guide more extensive public health responses, including vector management and enhancing epidemiological surveillance. Currently, the detection of specific IgM antibodies remains a cornerstone of diagnostic efforts, serving as a retrospective confirmation of EEE infection [15,25]. The continued development and refinement of diagnostic assays are vital not only for improving patient outcomes but also for informing public health policies aimed at reducing the incidence of this potentially devastating illness. As research advances, the integration of novel diagnostic modalities promises to enhance our ability to swiftly and accurately identify EEE virus infections, ultimately supporting more robust public health interventions.

## 4. Treatment Options and Public Health Implications

Current therapeutic approaches for Eastern equine encephalitis virus infection are limited and largely focused on supportive care rather than targeted antiviral treatment [1,32]. Patients with EEE often require intensive care, including respiratory support and seizure management, due to the severe neuroinvasive nature of the disease [1,32]. The disease’s rarity, combined with its high mortality rate, poses significant challenges to developing effective treatment protocols, especially in children [33]. Experimental approaches, such as the use of convalescent plasma, have shown some promise. However, these remain largely investigational and are not yet widely implemented due to a lack of robust clinical evidence supporting their efficacy. Public health strategies are pivotal in preventing EEE outbreaks, given the currently limited therapeutic options. Mosquito control measures are especially important in reducing vector populations [34]. Additionally, raising public awareness through educational campaigns about avoiding mosquito bites can significantly mitigate the risk of infection [35]. These strategies play a crucial role in high-risk areas and during peak transmission seasons. Importantly, ongoing surveillance programs are essential for early detection of viral circulation [35,36]. In light of these challenges, continuous research is essential for the advancement of both prophylactic and therapeutic solutions for EEEV. Vaccine development remains a top priority, aimed at providing long-term protection, especially for individuals in endemic regions. Alongside vaccine research, the exploration of novel antiviral agents offers hope for effective treatment options. As the scientific community advances towards understanding the virology and pathogenesis of EEE more thoroughly, these initiatives promise to enhance both individual patient care and broader public health outcomes in combating this devastating disease. 

## 5. The Relationship Between Human Metabolism and Viral Diseases

The intricate relationship between human metabolism and viral diseases has long been a subject of intense scientific scrutiny as researchers strive to unravel the complex interplay between the body’s physiological processes and the invasive nature of viral pathogens. Viral infections have been shown to adversely affect mitochondrial structure and functions, consequently impacting the metabolism and immune signaling pathways within the host [37]. Viruses have developed sophisticated mechanisms to target and manipulate these organelles, effectively hijacking the host’s metabolic machinery for their own proliferation and survival [38]. Mitochondrial dynamics, the delicate balance between fission and fusion [39], is often disrupted during viral infections, leading to an imbalance that can promote viral replication and facilitate the evasion of host immune responses [40]. Emerging evidence suggests that the convergence of various immune signaling pathways within the mitochondria is a critical factor in the activation, transcription, differentiation, and survival of immune cells [41]. Viruses, in turn, employ strategies to disrupt these mitochondria-mediated immune responses, further weakening the host’s defense and enhancing cell killing [42]. The impact of viral infections on human metabolism extends beyond the mitochondrial level, with potential implications for a wide range of viral diseases. Researchers have identified the crucial role of mitochondrial dynamics in the pathogenesis of severe acute respiratory syndrome coronaviruses, highlighting the need for a deeper understanding of the underlying mechanisms [43,44,45]. 

As the scientific community continues to delve deeper into the intricate interplay between human metabolism and viral diseases, the potential for groundbreaking discoveries and advancements in therapeutic interventions remains vast, with the promise of a more comprehensive understanding of these complex biological systems [37,42]. While the mechanisms by which viruses manipulate host cell metabolism and mitochondrial function are not yet fully understood, ongoing research has made significant strides in unraveling this complex relationship. Understanding the intricate interplay between metabolism and viral zoonosis holds the potential to inform the development of novel therapeutic interventions, targeting the metabolic vulnerabilities exploited by viruses to combat viral infections more effectively. 

## 6. Role of the NLRP3 Inflammasome in Viral Infections

This section aims to provide a comprehensive understanding of the intricate relationship between the Nod-like receptor protein 3 (NLRP3) inflammasome, viral infections, and mitochondrial dynamics, offering insights into the complex interplay between these biological processes. Inflammation underlying multiple diseases is closely associated with mitochondrial dysfunction, thus suggesting that targeting the inflammatory/mitochondrial axis may offer a therapeutic strategy to treat these diseases. The NLRP3 inflammasome has emerged as a critical pathophysiological mediator of inflammation. It is a multi-protein complex that is involved in pro-caspase-1 activation. Therefore, the NLRP3 inflammasome is a critical component of the innate immune system, playing a pivotal role in the body’s defense against various pathogens [46]. This protein complex is responsible for the activation of the inflammatory response, leading to the release of proinflammatory cytokines, such as interleukin-1β (IL-1β) and interleukin-18 [47]. Interestingly, the NLRP3 inflammasome has also been implicated in the regulation of mitochondrial function, which is a crucial aspect of cellular energy production and apoptosis [48]. The NLRP3 inflammasome is a well-known sensor of various pathogen-associated molecular patterns and damage-associated molecular patterns, including those derived from viral infections [49]. 

**Figure 2:**
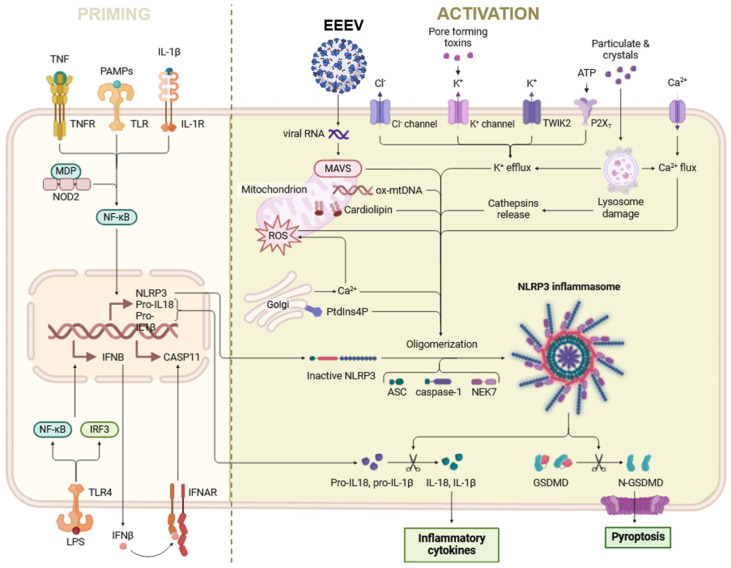
Scheme showing the interaction between EEEV, mitochondria, and the NLRP3 inflammasome. The left part of the scheme represents the priming step, and the right part of the scheme depicts the activation step of the NLRP3 inflammasome. The priming process leads to the activation of the transcription factor NF-κB and the subsequent transcription of canonical and non-canonical components of the NLRP3 inflammasome, whereas the activation process is responsible for the assembly of the NLRP3 complex and the subsequent release of inflammatory cytokines such as IL-1β and IL-18 and others. Priming starts once pathogen-associated molecular patterns (PAMPs) such as lipopolysaccharide (LPS) or cytokines (endogenous) activate the respective receptors on the cell membrane. The activation of the NLRP3 inflammasome during the viral infection upon viral RNA, proteins, and other components can directly or indirectly trigger the NLRP3 inflammasome, resulting in the release of inflammatory cytokines and pyroptosis.

Upon recognition of these patterns, the NLRP3 inflammasome undergoes activation, leading to the release of proinflammatory cytokines, which in turn initiate the host’s immune response to combat the viral infection [50]. This mechanism is particularly important in the context of respiratory viral infections, such as COVID-19, where the NLRP3 inflammasome plays a crucial role in the pathogenesis of the disease [51]. The activation of the NLRP3 inflammasome during viral infections has been extensively studied [51]. Viral RNA, proteins, and other components can directly or indirectly trigger the NLRP3 inflammasome, resulting in the release of inflammatory mediators that contribute to the clearance of the virus (Figure 2) [52]. However, the overactivation of the NLRP3 inflammasome can also lead to excessive inflammation, which may exacerbate the severity of the viral infection and cause [52]. Interestingly, the NLRP3 inflammasome has been found to be closely linked to mitochondrial function. Viral infections can disrupt mitochondrial homeostasis, leading to the release of mitochondrial DNA (mtDNA) and reactive oxygen species into the cytosol [53,54]. These mitochondrial-derived signals can then activate the NLRP3 inflammasome (Figure 2), further propagating the inflammatory response [47]. Conversely, the NLRP3 inflammasome can also influence mitochondrial function. The activation of the NLRP3 inflammasome has been shown to induce mitochondrial dysfunction, leading to the release of mitochondrial DNA and the generation of reactive oxygen species. This reciprocal relationship between the NLRP3 inflammasome and mitochondrial homeostasis highlights the intricate interplay between these two critical components of the innate immune system and cellular metabolism.

## 7. Relevance of Immunosenescence and Inflammaging for the Infection with EEEV

Age is one of several predictors that can impact the outcome of EEEV infection; juvenile animals appear to be particularly vulnerable to severe disease. This has also been observed in natural infections, as children are often the most severely impacted humans [55,56]. However, the underlying basis for this age-related susceptibility to severe illness in humans remains elusive. Interestingly, certain changes in the immune response to viral infections are often observed with age. As individuals age, their immune system undergoes a gradual but profound transformation known as immunosenescence. This phenomenon is characterized by a decline in the function of both the adaptive and innate components of the immune system, leading to increased susceptibility to infections, reduced vaccine efficacy, and a higher risk of chronic disease [57,58]. Immunosenescence is a complex process involving multiple interrelated mechanisms, including the above-mentioned metabolic dysfunctions, impaired immune responses to novel antigens, and inflammatory disorders [59]. Aging of the immune system is characterized by changes in the composition and function of T and B cells, as well as changes in the activity of macrophages, neutrophils, and natural killer cells [60]. However, a complete understanding of the mechanisms of immunosenescence remains elusive. Several of the latter mentioned have been identified, namely the involution of the thymus, reduced production and depletion of naive cells, altered ratios in subpopulations of immune cells (especially T cells), reduced quality of the adaptive immune response to antigens (cellular and humoral), dysfunction of hematopoietic stem cells (HSCs), and changes in cellular senescence of immune components [61,62]. Chronic inflammation related to age—inflammaging—was described for the first time in 2000 [63]. Today, we know that inflammation is, among other things, the result of forming a senescence-associated secretory phenotype (SASP), which is a direct consequence of cellular senescence. According to several authors, global inflammation creates favorable conditions for the development of age-related diseases [64]. It is noteworthy that EEEV-induced encephalitis is associated with significant changes in the expression of genes related to the activation of inflammation, which may be modified depending on the patient’s age. Therefore, research on vaccine development should also address this aspect. So far, vaccines have been tested in primates [16,65], and there is one report where immunoinformatics have been utilized to design a multiepitope vaccine candidate [66]. In the latter-mentioned study, the structural polyprotein of EEEV was analyzed by predicting T cell and linear B cell epitopes, and the vaccine construct had exceptional antigenic, nontoxic, and nonallergenic characteristics [65]. 

## 8. Conclusions

In summary, the Eastern equine encephalitis virus represents a significant public health challenge due to its intricate epidemiology and severe clinical manifestations. Its transmission, primarily through mosquito vectors, highlights the importance of vector management as a critical preventative measure. The confined geographical distribution in the United States, with heightened activity during late summer and early fall, further necessitates targeted vector control in vulnerable regions. The correlation of the EEE virus with immune-compromised individuals, particularly organ transplant recipients, necessitates enhanced screening protocols and public health strategies to minimize transmission risks in these susceptible populations. This piece further aimed to provide a better understanding of the intricate relationship between metabolism, the NLRP3 inflammasome, viral infections, mitochondrial dynamics, and aging, offering insights into the complex interplay between these biological processes to stimulate future research directions. There is an urgent need to develop effective EEEV preventive strategies.
